# Marburg virus disease outbreak in Kween District Uganda, 2017: Epidemiological and laboratory findings

**DOI:** 10.1371/journal.pntd.0007257

**Published:** 2019-03-18

**Authors:** Luke Nyakarahuka, Trevor R. Shoemaker, Stephen Balinandi, Godfrey Chemos, Benon Kwesiga, Sophia Mulei, Jackson Kyondo, Alex Tumusiime, Aaron Kofman, Ben Masiira, Shannon Whitmer, Shelley Brown, Debi Cannon, Cheng-Feng Chiang, James Graziano, Maria Morales-Betoulle, Ketan Patel, Sara Zufan, Innocent Komakech, Nasan Natseri, Philip Musobo Chepkwurui, Bernard Lubwama, Jude Okiria, Joshua Kayiwa, Innocent H. Nkonwa, Patricia Eyu, Lydia Nakiire, Edward Chelangat Okarikod, Leonard Cheptoyek, Barasa Emmanuel Wangila, Michael Wanje, Patrick Tusiime, Lilian Bulage, Henry G. Mwebesa, Alex R. Ario, Issa Makumbi, Anne Nakinsige, Allan Muruta, Miriam Nanyunja, Jaco Homsy, Bao-Ping Zhu, Lisa Nelson, Pontiano Kaleebu, Pierre E. Rollin, Stuart T. Nichol, John D. Klena, Julius J. Lutwama

**Affiliations:** 1 Department of Arbovirology, Emerging and Re-emerging Infections, Uganda Virus Research Institute (UVRI), Entebbe Uganda; 2 Department of Biosecurity, Ecosystems, and Veterinary Public Health, Collage of Veterinary Medicine, Animal Resources and Biosecurity, Makerere University, Kampala Uganda; 3 Viral Special Pathogens Branch, US Centers for Disease Control and Prevention (CDC), Atlanta, GA United States of America; 4 Kween District Health Team, Kween District Local Government, Kween, Uganda; 5 Uganda Public Health Fellowship Program, Ministry of Health, Kampala, Uganda; 6 African Field Epidemiology Network, Kampala, Uganda; 7 World Health Organization – Country Office, Kampala, Uganda; 8 Ministry of Health, Kampala, Uganda; 9 Public Health Emergency Operations Center, Ministry of Health, Kampala, Uganda; Institute of Tropical Medicine, BELGIUM

## Abstract

**Introduction:**

In October 2017, a blood sample from a resident of Kween District, Eastern Uganda, tested positive for Marburg virus. Within 24 hour of confirmation, a rapid outbreak response was initiated. Here, we present results of epidemiological and laboratory investigations.

**Methods:**

A district task force was activated consisting of specialised teams to conduct case finding, case management and isolation, contact listing and follow up, sample collection and testing, and community engagement. An ecological investigation was also carried out to identify the potential source of infection. Virus isolation and Next Generation sequencing were performed to identify the strain of Marburg virus.

**Results:**

Seventy individuals (34 MVD suspected cases and 36 close contacts of confirmed cases) were epidemiologically investigated, with blood samples tested for MVD. Only four cases met the MVD case definition; one was categorized as a probable case while the other three were confirmed cases. A total of 299 contacts were identified; during follow- up, two were confirmed as MVD. Of the four confirmed and probable MVD cases, three died, yielding a case fatality rate of 75%. All four cases belonged to a single family and 50% (2/4) of the MVD cases were female. All confirmed cases had clinical symptoms of fever, vomiting, abdominal pain and bleeding from body orifices. Viral sequences indicated that the Marburg virus strain responsible for this outbreak was closely related to virus strains previously shown to be circulating in Uganda.

**Conclusion:**

This outbreak of MVD occurred as a family cluster with no additional transmission outside of the four related cases. Rapid case detection, prompt laboratory testing at the Uganda National VHF Reference Laboratory and presence of pre-trained, well-prepared national and district rapid response teams facilitated the containment and control of this outbreak within one month, preventing nationwide and global transmission of the disease.

## Author summary

Marburg virus disease (MVD) is caused by the virus that belongs to the same family as that of Ebola Virus disease. The disease is characterized by severe clinical symptoms such as high fever, diarrhoea and vomiting, and severe bleeding from most body openings. On average, 54% of the people who get infected with Marburg virus die from it [1]. In October 2017, Uganda reported an outbreak of MVD in the eastern district of Kween that borders Kenya in the Mount Elgon plains. This was the first time MVD was being detected in this part of the country since previous MVD outbreaks had been reported in the western part of Uganda. The Ministry of Health together with partners instituted rapid outbreak response for control. Investigations revealed a cluster of one family involving four cases whereby only one survived. The outbreak was traced back into bat caves in Kween district. Rock salt mining in the cave led to a spill-over of the virus into the human population with the subsequent person-to-person transmission. Through contact tracing and isolation of the infected people among other approaches, the outbreak was brought under control as explained in this article.

## Introduction

Marburg Virus Disease (MVD) is a severe infectious disease caused by Marburg virus, a member of the *Filoviridae* family, which also includes Ebola viruses. Marburg virus was first recognized in 1967 when outbreaks of haemorrhagic fever occurred in laboratories located in Marburg and Frankfurt, Germany and Belgrade, Yugoslavia (present-day Serbia) [2]. MVD has a high case fatality rate ranging from 32% to 88% [1]. It is transmitted to humans after a spill-over event from a wildlife reservoir such as *Rousettus aegyptiacus* fruit bats or their faeces or contact with infected primates [3–5]. Human-to-human transmission occurs through direct contact with blood, body fluids, secretions and tissues of infected individuals or dead bodies. Marburg virus can be transmitted sexually and studies of Ebolavirus have shown that viral RNA can be detected in semen for up to 407 days [6]. The incubation period can last from 2–21 days and infected individuals are not viremic until initial symptom onset [7].

Three previous MVD outbreaks have been reported in Uganda [8–10]. The first recorded MVD outbreak was in 2007, in which three cases and one death were reported [8]. In 2012, a second MVD outbreak was documented, with a total 26 confirmed and probable cases, of which 15 (58%) were fatal [10]. This outbreak started in Ibanda District and subsequently spread to at least four additional districts including Mbarara, Kabale, Kamwenge and Kampala. A third outbreak was confirmed in 2014 involving a single MVD case identified in the capital Kampala [9]. Also, two additional outbreaks of MVD, one in the Netherlands and another in United States of America have been linked to Uganda [11, 12]

While the viral reservoir for Ebola Virus Disease (EVD) has not been definitively determined, one reservoir of Marburg virus has been shown to be *R*. *aegyptiacus* bats. *R*. *aegyptiacus* bats trapped in Kitaka mine and Python Cave located in the Albertine region of Western Uganda have been shown to be reservoirs of Marburg virus [4, 5, 13–15]. Following infection of Dutch and American tourists in 2007 and 2008, respectively, with Marburg virus after bat exposure at Python Cave [11, 12], investigators found that 2.5% of the *R*. *aegyptiacus* bats in this cave were Marburg virus -positive, using a viral specific PCR assay [4, 5]. Bats in Python Cave and Kitaka mine have been linked to four MVD outbreaks [8, 10–12]. Infected *R*. *aegyptiacus* bats do not appear to develop clinical symptoms or die as a result of infection with Marburg virus [15–17].

The Viral Haemorrhagic Fever (VHF) Surveillance and Laboratory Program located at the Uganda Virus Research Institute (UVRI) in Entebbe Uganda, received a blood sample from an individual suspected to be infected with a VHF virus from Kaproron Health Centre IV (Kween District) on 16^th^ October 2017. The sample was submitted by Kween District health team after suspecting a VHF following the death of two people in one family with similar clinical symptoms. The serum sample was tested on the day of arrival by RT-PCR for Crimean-Congo haemorrhagic fever virus, Ebolaviruses (*Bundibugyo ebolavirus*, *Sudan ebolavirus and Zaire ebolavirus)*, Marburg virus and Rift Valley Fever virus and found to be preliminarily positive for Marburg virus. The sample was re-tested and confirmed positive on 17^th^ October 2017. The Uganda Ministry of Health (MoH) National Task Force (NTF) was activated on 18^th^ October 2017 at the Public Health Emergency Operations Centre (PHEOC). A multi-sectoral and multidisciplinary National Rapid Response Team (NRRT) was established and deployed to Kween and Kapchorwa districts on 18^th^ October 2017. The outbreak was officially declared by the Ministry of Health (MOH) on 19^th^ October 2017. District Rapid Response Teams (DRRTs) were deployed within the affected Districts on the same day. This multi-sectoral team worked to conduct a rapid outbreak investigation and assessment, and initiated intervention measures. We present results from the epidemiological and laboratory investigations of the MVD outbreak and propose recommendations for future filovirus outbreaks.

## Methods

### Description of the outbreak area

**Fig 1:**
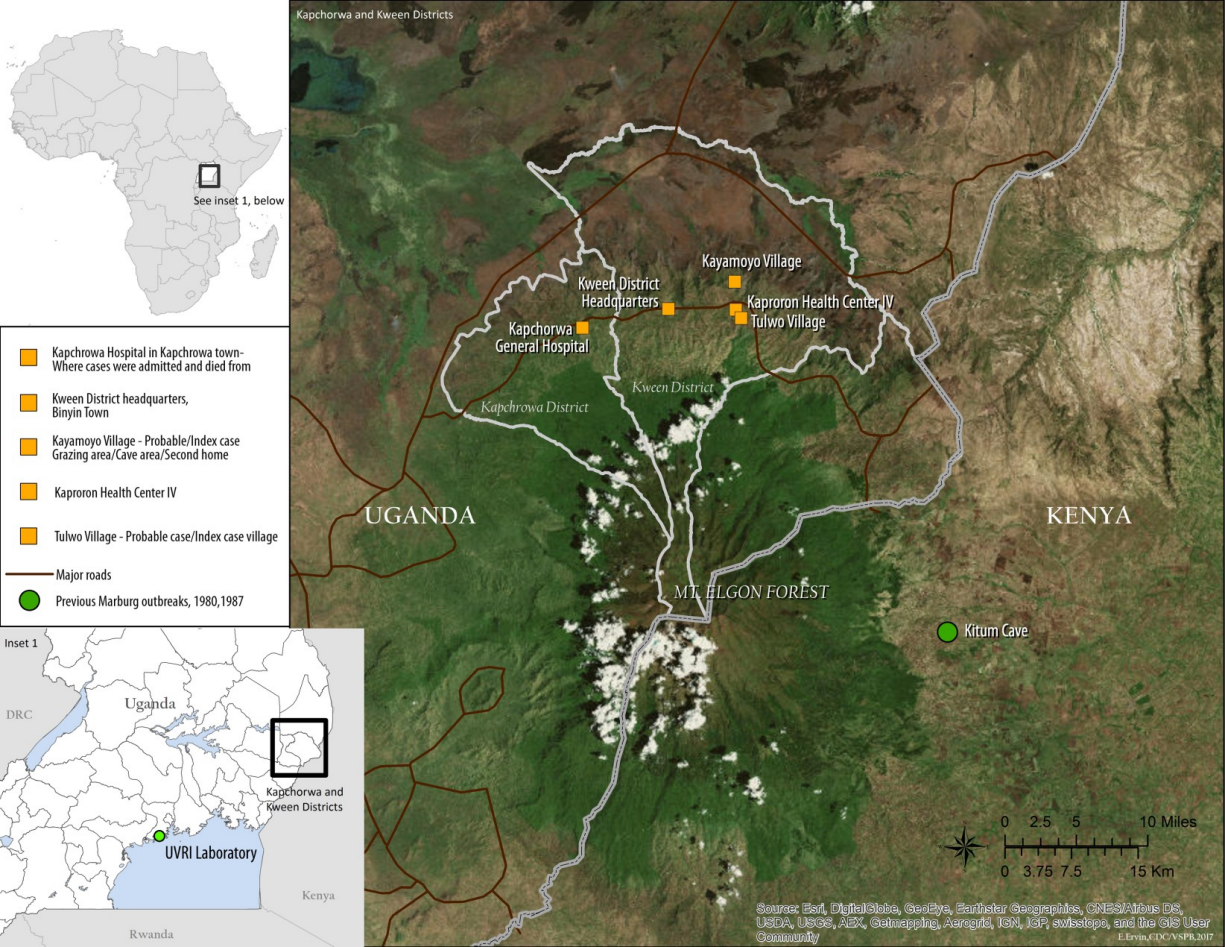
Map showing areas affected by the 2017 MVD outbreak. This figure was created specifically for this manuscript in ArcGIS using open source data from ESRI and DIVA-GIS for the background layers, and GPS points collected in the field for the points. (ESRI - http://opendata.arcgis.com/about, DIVA-GIS—http://www.diva-gis.org/).

Kween district was the epicentre of this MVD outbreak as all the cases lived and worked there. Kween district is adjacent to the Eastern border of Uganda with Kenya and is bordered by Kapchorwa district to the West, Bukwo district to the East, Nakapiripirit district to the north, Amudat district to the northeast, Bulambuli district to the northwest and the Republic of Kenya to the south (Fig 1). The district is located on the northern slopes of Mount Elgon, and has an average altitude of 1,900 metres (6,200 ft) above sea level. The estimated population of Kween district is 103,300 people (2012 census). District residents mainly engage in subsistence farming of food crops. The communities also engage in animal husbandry and raise a variety of livestock including cattle, sheep, and goats. The most common domestic animal is the donkey, which is often used for transport. The geography and mountainous terrain of Kween district are host to many bat-inhabited caves that are frequently visited by cattle keepers to collect “salt” rocks to feed their animals.

### Outbreak response team structure

The NRRT held initial briefings involving District Rapid Response Teams (DRRT) from Kween and Kapchorwa districts after which District Task Forces were activated. Kapchorwa district was included in this response, all confirmed cases sought treatment at the Kapchorwa district hospital, having been referred from the neighbouring Kween district. Four specialised teams (sub-committees) were formed and included; case management and infection control, surveillance and laboratory, social mobilization, and response coordination. The sub-committees were tasked to implement key interventions for the response and obtain clinical, epidemiological, laboratory, socio-cultural, ecological and socio-cultural data in order to better characterize the MVD outbreak in the two affected districts. Each specialised team was comprised of subject matter experts from the national and district levels that engaged in the key activities detailed in the sections below. Each team was led by personnel from the respective districts.

### Case finding

Clinicians working with epidemiologists and laboratory experts reviewed the clinical notes and preliminary investigations undertaken on the MVD cases admitted to the health facilities of Kaproron Health Centre IV (Kween district) and Kapchorwa Hospital (Kapchorwa district). Patient case histories and physical examination findings were extracted with all the essential clinical and epidemiological information captured onto the national VHF case investigation form. To facilitate the identification of additional cases, as in previous outbreaks, a working MVD case definition [9, 10] was used to classify cases as either suspected, probable or confirmed. A suspected case was defined as any person meeting one or more of the three following criteria: 1) Fever (≥37.5°C axillary body temperature) and sudden onset with three or more of the following symptoms: loss of appetite, headache, vomiting, abdominal pain, diarrhea, intense fatigue, myalgia and/or joint pains and history of contact with patient with similar symptoms; 2) Sudden onset sickness with or without fever and unexplained bleeding from any of the following sites: gastrointestinal tract (blood in vomitus), gums, nose, eyes genital (non-menstrual) and any other body site; 3) any unexplained and/or sudden death. A probable case was defined as any suspected case with an epidemiological link to a confirmed case. A confirmed case was defined as any person with either a positive PCR, IgM or IgG ELISA laboratory result for MVD, including retrospectively identified cases.

The suspected case definition was disseminated to health facilities in the affected and surrounding districts. Similarly, radio programs were conducted to sensitize the public on the symptoms of an MVD case, possible virus transmission routes and steps to control the spread of the disease. An alert desk equipped with contact information for the District Surveillance Officer (DSO) was established to receive and coordinate verification of alerts.

### Environmental and ecological investigations

An ecology team was assembled comprising of District Veterinary Officers (DVOs), Animal Husbandry Officers, the District Natural Resource Officer, the UVRI VHF program team and other DHT members. Initial investigations centred around the community of Kaptum grazing grounds, Kween District, where the initial reported case resided before falling ill. The team performed an environmental assessment of the grazing grounds using a snowballing approach (e.g., community members were asked to identify activities of the initial probable case, including caves he visited, in the month before becoming ill) and conducted interviews with local community and family members of the initial probable case. Caves were identified in the vicinity of this community used by residents for salt mining. The team investigated these caves to look for the presence of the known Marburg virus reservoir host, *R*. *aegyptiacus* bats.

### Laboratory procedures

Three to five millilitres of blood were obtained from all suspected cases of MVD for laboratory testing using real time RT-PCR and ELISA at the UVRI VHF laboratory according to established protocols [18, 19]. Briefly, RNA was extracted from whole blood using 5X Magmax™ 96 Viral Isolation kit (Applied Biosystems Inc., Vilnius, Lithuania) according to manufacturer’s instructions. Subsequent RT-PCR assays targeted the VP40 viral gene. ELISA for anti-Marburg IgM and IgG detection was performed using 96-well plates. Unless otherwise stated, all ELISA procedures used 100μl test volume per well format; plates were washed 3 times using 0.1% Tween-20 in PBS (v/v) between all procedures; and all incubation temperatures were at 37°C for 1h. In addition, all reagents used in all procedures were diluted in PBS containing 5% skimmed milk (also called serum diluent). To perform ELISA, all plates were pre-coated overnight at 4°C with Marburg antigens (for IgG) and an anti-human IgM (mu) antibody (for IgM) in physiological buffered solution (PBS). Samples were pre-diluted to 1:100 in serum diluent before their addition onto pre-coated plates. For IgM, the addition of samples onto plates was followed by the addition of a positive antigen (1:2 dilution) on one (upper) half of the plate and a mock antigen (1:2) on the other half (lower) of the plate. This was followed by the addition of a primary antibody, a rabbit anti-Marburg antibody (in a dilution of 1:1500) and then a secondary, horseradish peroxidase conjugated, antibody (in a dilution of 1:8000). The substrate used was 2,2′-azino-bis (3-ethylbenthiazoline-6-sulfonic acid (Kirkegaard and Perry Laboratories, Gaithersburg USA) read at 410nm. For IgG antibody detection, samples were added to precoated plates followed directly by an anti-human IgG conjugate. Similarly, to IgM, the substrate used was 2,2′-azino-bis (3-ethylbenthiazoline-6-sulfonic acid (Kirkegaard and Perry Laboratories, Gaithersburg USA) read at 410nm.

Samples collected during the investigation were transported to UVRI using designated vehicles for transportation of specimens, coordinated by the Uganda Central Public Health Laboratory (CPHL) in Kampala. A total of 34 individual blood samples were investigated as suspect cases of MVD. Additionally, 36 blood samples from previously identified close contacts of the initial probable case, suspected to be the index case, of this outbreak. These contacts were not actively monitored through contact tracing and had already passed the 21-day follow-up period prior to confirmation of the outbreak. All samples were sent to the laboratory to assess for evidence of previous or active infection using serological and molecular testing. Any acutely positive samples identified were later sent to the Viral Special Pathogens Branch (VSPB) laboratory at CDC in Atlanta, GA, USA for secondary confirmation testing, sequencing and isolation.

#### Next generation sequencing and bioinformatics analysis

Plasma (500 μL) and blood (150μL) specimens collected from first two confirmed cases, were inactivated with Tripure (Roche, Basel Switzerland). RNA was extracted after phase separation and applied to Clean and Concentrate-25 (Zymo Research, California United States) columns for further purification and concentration. RNA was treated with RNase-free DNase (Roche) and prepared for unbiased next generation sequencing (NGS) using the TruSeq RNA Access Library preparation kit with filovirus-specific enrichment oligos [20]. NGS libraries were also prepared using rRNA depletion and NEBNext Ultra II Directional RNA library preparation kit (NEB) [21]. Libraries were sequenced using an Illumina MiSeq (V2) and MiniSeq (2 × 150 cycles). Using custom scripts, Marburg-specific reads were mapped to KP985768 after depletion of adaptors (cutadapt) and quality trimming (printseq-lite). Average de-duplicated coverage was 2,781 and 9,092. The evolutionary history was inferred using all available full-length Marburg and Ravn genomes from Genbank using phyml (GTR+Γ(n = 4)–s SPR) with bootstrap support provided by 1000 iterations. Marburg genomes were deposited to Genbank: MH638314-5.

### Contact identification and follow-up

All contacts of the confirmed and probable cases were identified and listed for follow-up to ensure timely identification and isolation of new cases. Contacts with confirmed or probable cases from their date of symptom onset were defined as people who: 1) touched the body fluids of a case (blood, vomit, saliva, urine, faeces; 2) had direct physical contact with the body of a case (alive or dead); 3) or shared the linens, clothes, or dishes/eating utensils of a case and 4) slept, ate, or spent time in the same household or room as a case.

All contacts listed were followed up on a daily basis for 21 days after the last exposure to a probable or confirmed case. Under the supervision of the DSO, contacts were followed-up by trained Village Health Team (VHT) members and/or health workers who in turn submitted daily information detailing the health status of contacts under follow-up. The information collected by VHT members included temperature and MVD symptoms. All contacts under follow-up were advised to remain at home and to report any febrile illness to the designated VHT members or health workers. Any contact developing febrile illness during the follow-up period was reported to the alert desk using a dedicated phone number of the DSO. All contacts completing the 21-day follow-up period without developing disease symptoms were dropped from follow-up and encouraged to resume their normal daily routine.

All suspected and confirmed cases were treated in designated cubicles in the MVD treatment units and started on supportive treatment including intravenous fluids, correction of electrolyte imbalances, and treatment for secondary infections. Strict barrier nursing and infection, prevention and control measures were observed in the treatment facility. On-the-job training in personal protective equipment (PPE) and infection prevention and control measures were provided for local healthcare providers.

### Contacts of initial probable case

During initial epidemiological investigations, many of the identified contacts of the initial probable case were found to have already passed the 21-day follow-up period. In order to ensure no unidentified cases or transmission chains were occurring in the community prior to the investigation teams’ arrival in the district, blood samples were collected from close contacts as defined above. Similar investigations were carried out during previous filovirus outbreaks in Uganda where confirmed cases were retrospectively identified through serological testing of contacts of confirmed and probable cases and initially missed by either surveillance prior to outbreak investigation or during the outbreak [10]. A total of 36 close contacts of the initial probable case were sampled for serological and molecular testing.

### Data management and analysis

All data collected from cases and contacts were managed using the VHF EpiInfo application [22] by the district biostatisticians, supported by NRRT members. The VHF EpiInfo software has been previously used to manage MVD outbreaks in Uganda in 2014 [9] and was also adopted for data management during the 2014–15 West Africa EVD outbreak [22]. This software has a number of useful assets with respect to VHF outbreak response including: (1) ensuring that the correct follow-up period is observed for each contact; (2) generating daily follow-up lists for active case contacts and updating their status after follow up; (3) linking contacts with their known source cases; (4) built-in epidemiological data analysis for real-time situational awareness; (5) removal of contacts from the follow-up list once they have completed the 21-day follow-up or once the source case to which they are linked tests negative (6) capability to generate transmission chains based on case linkage information; and (7) implementation of a large number of complex business rules associated with contact tracing without needing user intervention, thus lessening the amount of data entry errors and mistakes. All case and contact data were entered in the VHF EpiInfo application where real-time analysis was carried during the investigations and presented at the daily district task force update meetings. Data was later exported into STATA software for further analysis. Probable and confirmed cases were compared with suspected cases that tested negative (controls) in respect to clinical cases and risk factors using Fisher’s Exact test was used to compute a p-value and statistical significance was considered as a p-value ≤ 0.05.

### Ethics statement

This outbreak investigation is a public health emergency that was approved by the National Task Force (NTF) for disease outbreaks in Uganda, and hence it was considered a non-research activity. All persons who were interviewed and whose samples were tested gave informed oral consent, and a parent or guardian of any child participant provided informed consent on the child’s behalf.

## Results

### Epidemiological description of the outbreak

#### Description of the cases

**Fig 2:**
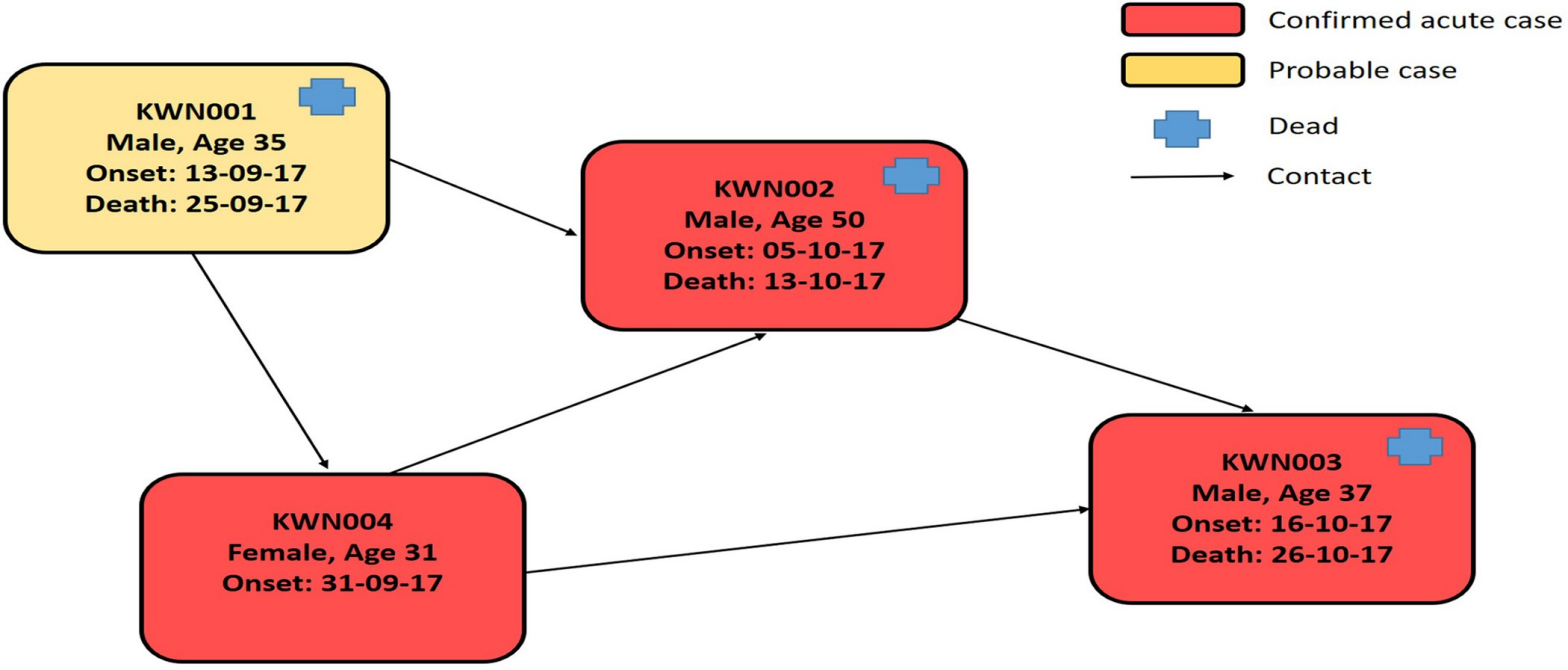
Transmission chain for MVD cases, Kween District, 2017.

**KWN001**: The initial probable index case was a 35-year-old male herdsman (cattle and goats) from Tulwo village, Kamwam Parish, Kaproron Sub-county of Kween District who undertook frequent hunting forays to the Kaptum grazing grounds in Kween District, which include a cave inhabited by bats of *Rousettus* species. The investigation established that he was also a crop farmer. He lived in an area close to a cave, inhabited by *Rousettus* species fruit bats, on the slopes of Mount Elgon. Contacts reported he frequented the interior of the cave with children to mine rock salt for livestock and collect bat droppings for manure for his crop farm. Onset of his symptoms began on 13^th^ September 2017 for which he was treated at a local private clinic at Cheminy ward in Kaproron Town Council on 20^th^ September 2017. He presented with complaints of high fever, vomiting, diarrhoea, intense fatigue, abdominal pain, muscle pain, headache, joint pain and hiccups. The patient was referred to Kapchrowa Hospital on 23^th^ September 2017 when his condition deteriorated, with hematemesis, convulsions and unconsciousness. He died on 25^th^ September 2017 and was buried on 27^th^ September 2017 following routine funeral rites according to family norms which involved washing and dressing the body, putting the body in a coffin, all handled by close relatives some of which became infected with MVD (KWN002 and KWN003-Fig 2). No samples were collected for laboratory investigations. A total of 36 contacts of this initial probable case were listed, although by the time of investigation the mandatory 21-day follow-up period for monitoring these contacts had already elapsed. The investigation team collected blood samples from this patient’s close contacts to investigate the possibility of undetected MVD infection with Marburg virus. One specimen from this group of contacts was serologically positive for MVD (KWN004), for whom additional description is provided below. KWN001 had no history of exposure to a known suspect case or ill person, no history of participating in a funeral, and no history of hospitalization or travel outside the village in the one-month preceding onset of his illness. Despite being a hunter, family members reported that he had not hunted during the four weeks preceding illness onset. However, his family reported that he frequently entered caves inhabited by bats to mine cattle salt licks, an activity he had done three weeks before developing symptoms consistent with MVD.

**KWN002:** This was the first laboratory-confirmed case from the outbreak and was a sister to KWN001. She was a 50-year-old housewife who nursed KWN001 during his illness in Kapchorwa hospital and participated in cultural burial rituals that included cleaning of the dead body of KWN001. Subsequently, she became ill and was admitted to Kaproron Health Centre IV, Kween District, on 5^th^ October 2017 with fever and bleeding. On 10^th^ October 2017, she was referred to neighbouring Kapchorwa District Hospital. She died on 13^th^ October 2017 and had a supervised burial on the same day in Chemuron Village, Kween District. However, because she was a sister and caretaker of KWN001 and presented with similar clinical symptoms, the Kween District Health Team (DHT) had a high index of suspicion for a VHF infection. A blood sample was collected from the dead body during the supervised burial and was sent to the VHF laboratory at UVRI for testing. The blood sample was tested on 17^th^ October 2017 for a panel of viruses causing haemorrhagic fever by RT-PCR and tested positive for Marburg virus using three independent, RT-PCR assays, confirming Marburg virus infection. The sample was subsequently shipped to the VSPB laboratory of CDC in Atlanta for further testing and genetic analysis and secondary confirmation by RT-PCR. Subsequently, Marburg virus whole genome sequencing was performed directly from the clinical specimen. Because KWN002 was not managed in an isolation unit in the two health facilities where she was admitted, there was concern for potential exposure and infection of health care workers and caretakers. A total of 87 contacts were listed and followed up for 21 days, one of them (KWN003) became a case of MVD after testing positive Marburg virus.

**KWN003:** The second laboratory confirmed case in this outbreak; was a 37-year-old male who was a brother to both the probable and confirmed cases, KWN001 and KWN002. He was listed as a contact of the two previous cases and was reported to have developed clinical symptoms on 16^th^ October 2017, which became progressively severe and was very ill by 22^nd^ October 2017. His condition deteriorated, and he was admitted to the MVD treatment unit with fever, body weakness, abdominal pain, lack of appetite, joint pains and history of hematemesis while at home. He was given supportive treatment but died on 26^th^ October 2017 after experiencing profuse “coffee ground” hematemesis. A blood sample tested positive for Marburg virus at UVRI by RT-PCR. The case management team conducted a safe burial amidst protests and violence from community members who did not believe a member of their community had died of MVD. A total of 101 contacts were listed for this case, none of whom developed MVD illness.

**KWN004:** The third laboratory confirmed case was a 31-year old female and the second wife of the initial probable case, KWN001. This case was identified retrospectively from testing close contacts of KWN001 who had already passed the 21-day follow-up period at the start of the epidemiological investigation. KWN004 had also participated in the burial of the KWN002, her sister-in-law. Her reported date of symptom onset was 30^th^ September 2017 and she sought treatment in a private health facility in Kapchorwa town on 3^rd^ October 2017. She continued feeling sick and had a miscarriage on 5^th^ October 2017 with additional symptoms of headache, vomiting, intense fatigue, abdominal pain and bleeding. She recovered from the illness but remained weak. A blood sample collected on 20^th^ October 2017 was sent to UVRI for testing and was positive for Marburg IgM and IgG antibodies by ELISA. No specific contacts were listed for this case as it was confirmed retrospectively after 21 days of follow-up had passed. However, her contacts were the same as those of KWN001 (her husband) and family members (KWN002 and KWN003).

By the end of the outbreak response (8 December 2017), 70 persons had been epidemiologically investigated and laboratory tested for MVD. Only four cases (5.7%) were categorized as having MVD including one probable case and three confirmed cases. The three laboratory confirmed cases were classified as acute based on RT-PCR and/or IgM ELISA positive results (Fig 2). Of the four MVD cases, three died (two confirmed acute and one probable), yielding a case fatality rate of 75% for the outbreak. Fifty percent (2/4) of the four cases were females, and the age range was 31–50 years. None of the confirmed or probable MVD cases was a healthcare provider, although all of the deaths occurred in a healthcare facility. All the three confirmed cases reported fever, vomiting, and abdominal pain and haemorrhage as clinical symptoms (Table 1). Of the thirty-four people tested, thirty-two had fever and at least three other clinical symptoms, two had no fever but had unexplained bleeding from one of the body orifices and no unexplained deaths were seen during this investigation. Of the thirty-six contacts of the initial probable case, only one was found positive by IgM/IgG testing (KWN004).

**Table 1:** Clinical symptoms of confirmed and probable cases compared to suspect cases that tested negative for Marburg virus.

Symptom	MVD Cases (n = 4)	Non-MVD cases (n = 30*)	p-value
**Fever**	4 (100%)	25 (86.5%)	0.56
**Vomiting**	4(100%)	14 (58.3%)	0.1
**Diarrhoea**	2 (50%)	8 (33.3%)	0.29
**General Body Weakness**	4 (100%)	15 (68%)	0.5
**Anorexia**	4 (100%)	10 (47%)	0.05
**Abdominal Pain**	4 (100%)	15 (60%)	0.26
**Chest Pain**	1 (25%)	4 (18%)	0.75
**Joint Pain**	4 (100%)	5 (24%)	0.004**
**Headache**	3 (75%)	17 (65%)	0.7
**Cough**	2 (50%)	7 (28%)	0.3
**Difficult Breathing**	1 (25%)	2 (8.7%)	0.36
**Difficulty Swallowing**	1 (25%)	0 (0.0%)	0.14
**Sore Throat**	1 (25%)	13(14%)	0.5
**Jaundice**	2 (50%)	1 (6%)	0.048**
**Conjunctivitis**	2 (50%)	1 (4%)	0.27
**Skin Rash**	1 (25%)	2 (8%)	0.3
**Hiccups**	2 (50%)	0 (0.0%)	0.01**
**Any Unexplained Bleeding**	4 (100%)	12 (44%)	0.04**

*denominator varies per variable because of missing information.

** statistically significant

#### Contact tracing

**Fig 3:**
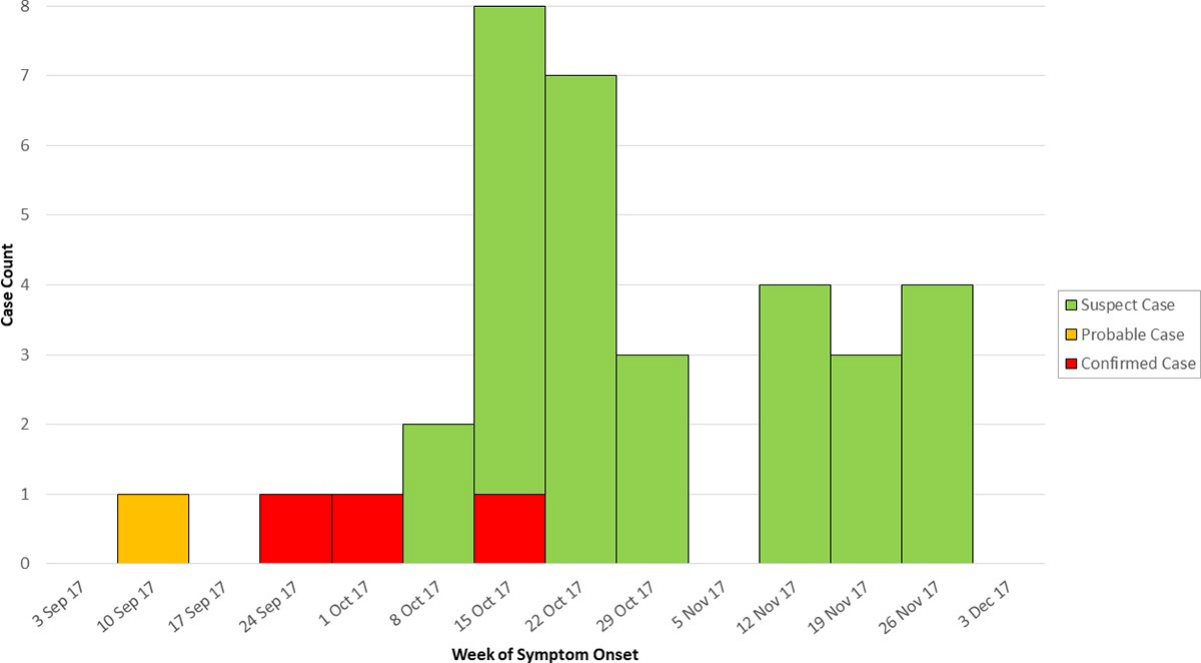
Epidemic curve of the MVD outbreak in Kween District between, September 10 to December 10, 2017.

A total of 299 contacts were listed and followed-up; 225 resided in Kween district, 41 in Kapchorwa district, 13 in other districts of Uganda, 19 from Kenya and 1 from an unspecified location. Eight (8) contacts developed symptoms of suspected MVD during the follow up period during the outbreak investigation. Only one contact tested positive and was confirmed MVD, KWN003. The outbreak started on 16^th^ September 2017 and ended on 8^th^ December 2017 (Fig 3). The most significant risk factor for infection with Marburg virus in this outbreak was close contact with a confirmed case (p-value = 0.021) and attending a funeral (p-value = 0.07). Other factors such as sex, age and occupation did not significantly affect the outcome of developing MVD (Table 2).

**Table 2:** Epidemiological risk factors for being MVD cases.

Risk Factor	MVD Cases (n = 4)	Non-MVD cases (n = 30*)	p-value
**Contact with a Case**	3 (75%)	4 (13%)	0.021**
**Attending a Funeral**	3 (75%)	2 (7%)	0.007**
**Male Sex**	2 (50%)	16 (53%)	0.9
**Travel Before Illness**	1 (25%)	3 (10%)	0.85
**Farmer**	1 (25%)	8 (27%)	0.9
**Hunter**	1 (25%)	0 (0%)	0.11
**House Wife**	2 (50%)	4 (13%)	0.09

*denominator varies per variable because of missing information.

** statistically significant

### Phylogenetic analysis

**Fig 4:**
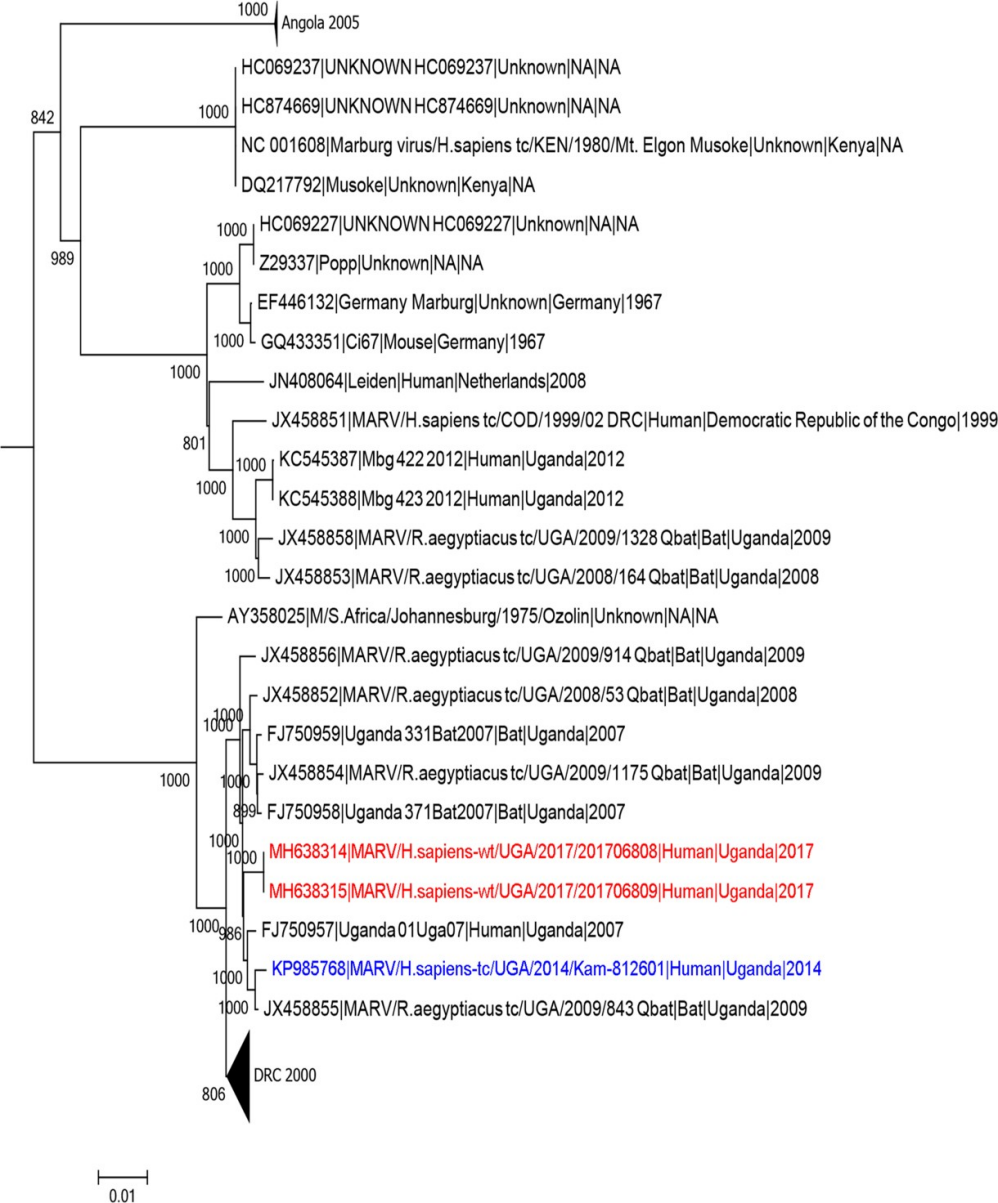
Phylogenetic tree inferring the relationship of Marburg virus from the 2017 outbreak with previous MVD outbreaks in Uganda.

The full genomic sequence of MARV (MBG201708608 and MBG201708609, Fig 4, red) falls into a cluster that consists of MARV sequences isolated from humans and bats in Uganda between 2007–9 and 2014 (Fig 4, blue) but are distinct from Marburg virus sequences collected during a previous outbreak in Kabale, Uganda in 2012 (Fig 4, KC545387 and KC545388). The 2017 Marburg virus sequences share a recent common ancestor with other Marburg virus sequences collected from Uganda. These include a human MVD case detected in 2014 living in Kampala and Kasese districts (KP985768), a virus sequence collected from a miner who worked in the Kitaka mine in July 2007 (3,7) and from virus sequences obtained from bats that were collected from either Python Cave or the Kitaka mine during 2007–2009. The sequences from the 2017 cases were indistinguishable from each other, suggesting person-to-person transmission.

### Environmental and ecological findings

**Fig 5:**
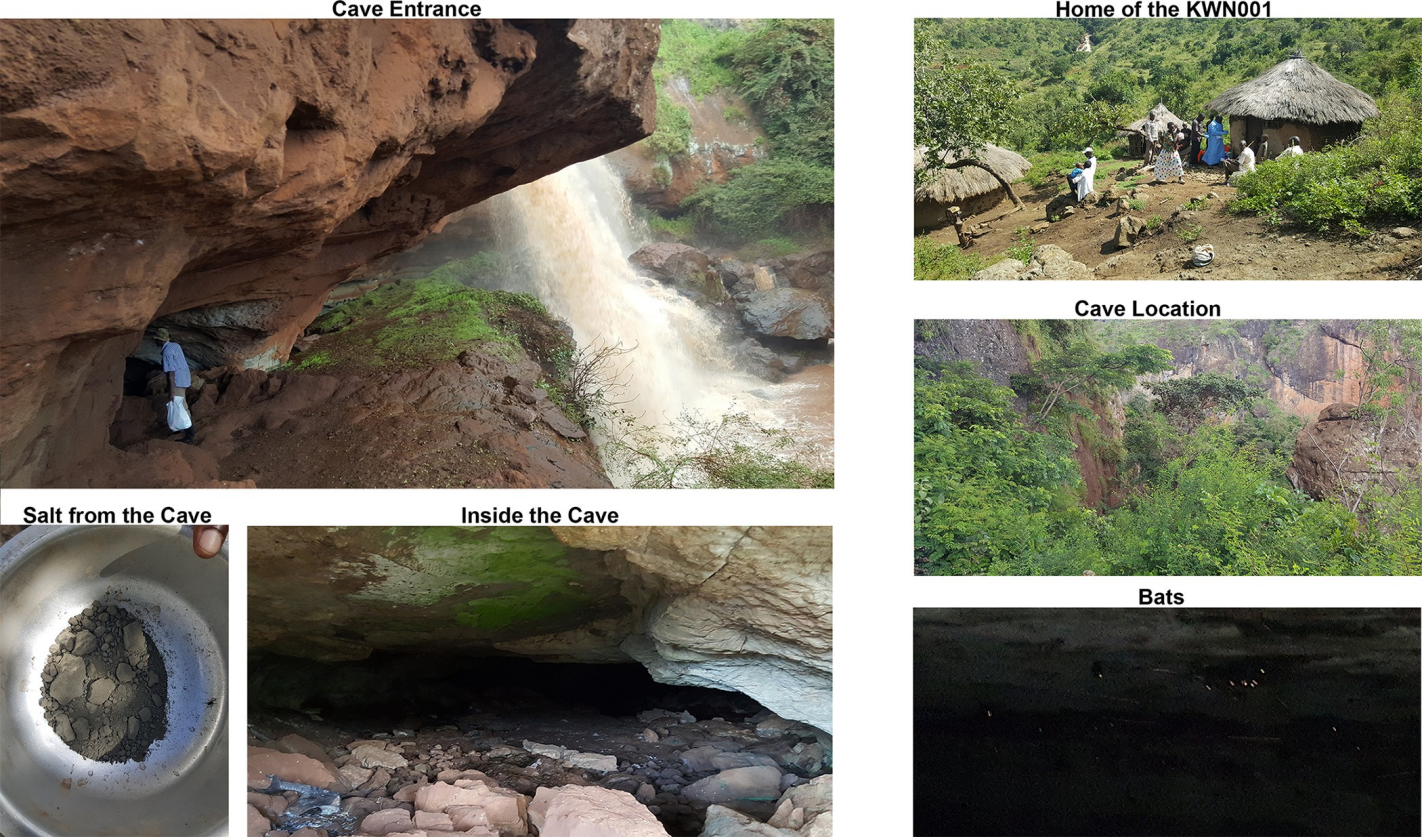
Kaptum cave and gopil falls on the sundet river in Kween District, home of the probable case, salt mined in the cave and bats of *Rousettus* species inside the cave (credit: luke nyakarahuka).

The bats inhabiting the caves visited in Kween district were identified as *Rousettus* species. However, the ecological investigation team was unable to conclusively identify the bats as *R*. *aegyptiacus* since trapping of the bats was not performed. Gross visual and physical identification supports the *R*. spp. identification. Additional ecological studies are needed to identify the species of *Rousettus* observed *and* confirm Marburg virus presence in the bat population inhabiting these caves (Fig 5).

## Discussion

We describe here the first MVD outbreak reported in Eastern Uganda [8]. Previous MVD outbreaks in Uganda have been linked to Kitaka and Python caves in Western Uganda [4, 11, 12], and one MVD outbreak, detected in Central Uganda, which involved a single case in 2014 [9]. The four cases of this outbreak in Kween district, which involved one cluster of closely-related family members and did not spread to other districts within or outside Uganda. This could be attributed to enhanced capacity of the Uganda National VHF and other MOH surveillance structures and their capability to quickly identify, detect and respond to filovirus outbreaks as has been described previously [23, 24].

As with other MVD outbreaks in Central and Eastern Africa [25–27], the initial case in this outbreak, KWN001, appears to have been linked to activities taking place within a cave harbouring *Rousettus* bats that are a known reservoir of Marburg virus [4, 5, 13, 15]. KWN001 lived and worked in an area surrounded by cliffs within the Mt. Elgon Forest Reserve range, approximately 800m from a cave inhabited by these bats. It is possible that mining salt and collection of manure from the caves exposed KWN001 to excreta and/or other Marburg virus-contaminated material from these bats, and that this is the most probable initial source of virus exposure in this outbreak.

While no previous outbreaks of MVD have occurred in this region of Uganda, *R*. *aegyptiacus* habitat has been well-documented throughout the Mt. Elgon region [28], and a recent filovirus risk mapping exercise has shown that this region is at risk of MVD outbreaks [29].

However, many questions remain regarding the exact mechanism behind spillover events of Marburg virus from the wildlife reservoir to human population. One possible explanation has been the observation that Marburg virus is shed in saliva more than through other routes [15]. Residue of bat saliva on fruits such as mangoes, guavas, and apples can cause a spillover event into humans who may consume these foods without properly washing them. However, in this particular outbreak, we believe that the most likely spillover event occurred as a result of salt mining and manure collection activities performed by KWN001 in a cave occupied by *Rousettus* spp. bats. Small injuries sustained by KWN001 from pounding rocks contaminated with urine, saliva and feces of bats may have contributed to exposure and infection. Another potential risk factor for KWN001 was direct exposure to bats via hunting, but it remains uncertain if he performed this activity.

The observed case-fatality rate (CFR) of 75% is higher than what has historically been reported in other parts of Uganda. Apart from outbreaks with single fatal cases [9, 11, 25, 30], most outbreaks of MVD have reported CFRs of below 50% [8, 10, 31–33]. Only two outbreaks have reported a large number of cases; the first occurred in the Democratic Republic of the Congo (DRC) in 1998 where 154 cases were recorded, and the second occurred in Angola in 2004 with 254 recorded cases. These outbreaks had CFRs of 83% and 90%, respectively [26, 27]. Although the CFR in the Kween outbreak is considered high, the rapid recognition of a MVD outbreak and epidemiological response could have resulted in the small number of cases. The high CFR seen in this outbreak might be attributed to the cases seeking healthcare late in the course of illness, at which point supportive care is typically less effective [34]. It is also believed that the all four initially sought treatment from traditional healers, and only later sought care from local clinics, at which point their illness was more severe. Additionally, the high CFR in this outbreak may be attributed to other host factors such as immunological status at the time of infection and host genetic factors since all fatalities were in related family members. Further analysis into other factors, such as viral genetic attributes or route of transmission, contributing high CRF should be investigated.

Unlike previous outbreaks of MVD in Uganda in 2007 and 2012 [8, 10], clinical symptoms of MVD in this outbreak were slightly different and more severe with all the four cases exhibiting fever, vomiting, abdominal pain, general body weakness, anorexia and joint pain (Table 1). While not always present early during filovirus infections, bleeding is a common symptom associated with disease progression. In this outbreak, symptoms of bleeding from body orifices were more prevalent than in other filovirus outbreaks, where bleeding symptoms are usually observed in 50% or less of the cases. Additionally, hiccups, which are highly associated with filovirus infections [10], were found in 50% of cases.

Evidence from recent outbreaks of MVD and Ebola Virus Disease (EVD) suggests that a minority of infected individuals may develop mild infections that would not otherwise be detected through standard VHF surveillance or outbreak investigation methods [10, 35]. In one such instance in the October 2012 outbreak of MVD in Kabale, Southwestern Uganda, an investigation identified retrospective cases through serological analysis that suggests the outbreak had actually begun in early June 2012 in neighboring Ibanda district, and not in the Kabale area where the previously-identified initial case was identified [10]. In 2011 during the investigation of an EVD outbreak in Luweero district, Central Uganda, one convalescent case was discovered in a family member of a PCR-confirmed case. This was an indication of past infection not acquired from the confirmed case. Additionally, the convalescent case did not recall having any illness similar to Ebola Virus Disease (EVD) in the past, suggesting that mild and undetected infection may have occurred [35]. Lastly, during a 2012 outbreak of EVD in Kibaale district, convalescent cases were retrospectively detected through serological testing of outbreak contacts that were followed but did not develop or report symptoms. Following on this experience, we collected samples from close contacts of the first probable case and identified one person that had both IgG and IgM for Marburg virus. These individuals had already passed their 21-day follow-up observation period recommended after contact with a filovirus case due to the first case being identified outside of this window of time. Therefore, when an outbreak begins after a majority of contacts have passed their 21-day follow-up periods, the testing of close contacts may be useful in the identification of unrecognized unreported cases, thus providing critical epidemiological information to characterize and describe the extent of the outbreak and identify additional contacts requiring follow-up.

Marburg virus sequenced from acute cases in this outbreak are indistinguishable (excluding missing coverage at the 5’ and 3’ gene segments), suggesting this outbreak resulted from a single spill over event with subsequent human-to-human transmission (Fig 4). This is further supported by the transmission chain demonstrating that close contact through nursing care and through participation in funeral rites; these activities were notable risk factors for Marburg virus infection in the epidemiology of the outbreak. Close contact with an infected person has been documented as a major risk factor in the transmission of filovirus during outbreaks. Additional research is needed to characterize the molecular epidemiology and ecology of Marburg virus in this region. We observed that the viral sequences from this outbreak in Eastern Uganda share a common ancestor with viral sequences from the 2014 MVD outbreak in Central Uganda involving a single case [9]. Conversely, the 2017 and 2014 sequences were distinct from the 2012 sequences collected in Southern Uganda (Kabale district) suggesting the presence of two unique Marburg virus populations. However, Marburg viruses isolated from populations of *R*. *aegypyiacus* bats residing in the same cave can contain sequences similar to these two Marburg virus populations and Ravn, thus making geographic associations of Marburg virus to the local bat populations extremely difficult. This point has been demonstrated in *R*. *aegypyiacus* bat populations captured in Kitaka and Python caves in Western Uganda [4, 13]. Additional research is under way to study the movement of *R*. *aegyptiacus* bats, determine their populations in Western Uganda and try to identify other populations they may interact with over large geographic areas in different regions of Uganda, and how this might impact the transmission of Marburg and other viruses. Given the presence of caves inhabited with *Rousettus* spp. bats throughout different regions in Uganda, and the human-bat interface within caves and the environment, there continues to be need for effective VHF surveillance throughout Uganda.

In conclusion, this was the fourth MVD outbreak to be laboratory-confirmed in Uganda, and the first in Eastern Uganda. Uganda was able to rapidly identify, confirm, respond and control the outbreak and prevent nationwide, and possibly international, spread of these viruses. This capacity has been developed over a period of 10 years since the 2007 Bundibugyo Ebola outbreak, after which enhanced VHF surveillance and laboratory detection was established in Uganda. The success of this outbreak response greatly contributed to have limiting the total number of MVD cases to only four. This again demonstrates how continued enhanced surveillance and rapid response for filovirus, as well as other VHFs and emerging zoonotic diseases, can contribute to global health security by controlling outbreaks at the source and preventing them from becoming global epidemics as was seen in the West Africa Ebola epidemic in 2014–15.
